# The Stereoscopic Anisotropy Is Smaller in Elderly Population

**DOI:** 10.1167/iovs.63.12.26

**Published:** 2022-11-17

**Authors:** Aracelis Aguilera-Francisco, Ignacio Serrano-Pedraza

**Affiliations:** 1 Department of Experimental Psychology, Faculty of Psychology, Universidad Complutense de Madrid, Campus de Somosaguas, Madrid, Spain

**Keywords:** stereoscopic anisotropy, stereoacuity, aging

## Abstract

**Purpose:**

The stereoscopic anisotropy is one of the most intriguing phenomena of stereoscopic vision. It shows that the disparity thresholds to detect three-dimensional sinusoidal horizontal corrugations are much lower than for vertical corrugations for spatial frequencies lower than 1 cycles/deg. A recent study has shown that the anisotropy increases during childhood and that visual experience probably plays an important role in its development (Serrano-Pedraza et al., 2016). Here we want to determine the impact that the visual experience has throughout life in the stereoscopic anisotropy.

**Methods:**

We performed two experiments testing two age groups of 35 participants each: the young group aged 18 to 45 years and the old group aged 62 to 90 years. We measured disparity thresholds for three-dimensional sinusoidal corrugations of 0.1 cyc/deg, with either vertical or horizontal orientation. Detection thresholds were obtained using Bayesian adaptive staircases. For each participant we computed the anisotropy index by subtracting the thresholds in logarithmic units of the vertical minus the horizontal corrugation.

**Results:**

The analyses show that stereo thresholds for vertical corrugations are similar for both groups, however, for horizontal corrugations the thresholds are much lower for the young group. Therefore, the anisotropy was much stronger in the young group (mean, 0.67 ± 0.46) than for the old group (mean, 0.24 ± 0.3). Pearson correlation between the anisotropy index and age shows a negative and significant correlation (*r* = −0.49; *P* = 1.83 × 10^−5^; *N* = 70), that is, as age advances, the anisotropy decreases.

**Conclusions:**

Thus, visual experience plays an important role in the development of stereo vision. Although disparity thresholds for horizontal corrugations in the older group are higher, surprisingly, disparity thresholds for vertical corrugations remain stable and do not change. Therefore, the stereoscopic anisotropy decreases with aging.

Binocular stereopsis is the process that allows us to judge depth from the small binocular disparities between the images of the world that are projected into both eyes. Given that our eyes are separated horizontally in our head, binocular disparities are mainly horizontal. Nevertheless, vertical disparities are also present and have an important role in depth perception.^1^^–^^5^ Interestingly, even if we restrict ourselves to horizontal disparities, there is a surprising and unexplained anisotropy in stereovision. The sensitivity to sinusoidal disparity corrugations (i.e., reciprocal of disparity thresholds), created by using random dot stereograms,^6^^,^^7^ is higher when they are horizontally oriented than when they are vertically oriented.^8^^–^^13^ In particular, sensitivity is higher for lower spatial frequencies (i.e., <0.4–1 cyc/deg).^8^^,^^10^ This anisotropy for low spatial frequencies is closely related to the anisotropy found using flat surfaces, where the sensitivity to surfaces rotated around the vertical axis (i.e., slanted surfaces that consist of compression disparities) is lower than to surfaces rotated around the horizontal axis (i.e., inclined surfaces that consist of shear disparities).^9^^,^^14^^–^^18^

Previous results have shown that, when representing the disparity thresholds for horizontally oriented sinusoidal corrugations^19^ as a function of the spatial frequency, the data show a U shape, similar to that found with luminance gratings.^20^ A similar U shape is also found for vertical corrugations, although disparity thresholds for low spatial frequencies are much higher. The lowest disparity threshold (i.e., maximum sensitivity) for horizontal corrugations occurs between 0.2 and 0.4 cyc/deg, and for vertical corrugations it is approximately 0.4 cyc/deg.^8^^,^^10^

Despite the strong evidence found about the stereoscopic anisotropy, little is known about the underlying mechanisms involved or its function. One possible explanation of this anisotropy is related to the size of the spatial summation fields in stereovision.^21^ These authors found that summation fields extend horizontally in space only for horizontal corrugations (they used three-dimensional [3D] Gabor patches) and the size of these fields increases with decreasing spatial frequency of the corrugation (aspect ratio 4:1 cycles). According to these authors, this property can be related with the statistical properties of natural images. Vertical contours (i.e., like branches) contain variations of luminance that support stereopsis; however, only texture supports stereopsis for horizontal contours, because these contours have no horizontal disparity. Thus, horizontally elongated summation fields are needed to summate the horizontal texture information and compensate for the difference between luminance and disparity information that is present in vertically and horizontally oriented edges of natural images. Therefore, this compensation mechanism predicts that disparity thresholds for low spatial frequencies, when only one cycle is visible, are lower for vertical corrugations than for horizontal ones. However, we found opposite results using a very low spatial frequency (0.05 cyc/deg) with only one cycle visible^10^ (see Fig. 2B) (i.e., disparity thresholds for vertical corrugations are higher than for horizontal corrugations).

An alternative explanation of the stereo anisotropy was proposed by our laboratory. We suggested that one spatial frequency stereo channel could be responsible for the detection of vertically oriented corrugations and that more than two spatial frequency stereo channels are responsible for the detection of horizontal corrugations.^10^ However, in a different study, we tested this hypothesis using a masking paradigm and found multiple channels for detecting both vertical and horizontal corrugations.^12^ Other authors, using a different type of stimulus and different procedures, also found multiple disparity channels for detecting both orientations.^22^^,^^23^ Therefore, the hypothesis of a single disparity channel cannot be sustained.

In an interesting article,^11^ the authors measured the stereoscopic anisotropy in barn owls and found the opposite anisotropy (i.e., higher sensitivity for vertical than for horizontal corrugations). They suggested that the brain promotes the disparity gradients that are present in natural images when they are behaviorally relevant. However, this notion opens the question of whether this anisotropy develops with practice or is present from birth. Our laboratory tested this hypothesis and found that the stereo anisotropy is stronger for adults than for children, suggesting that visual experience plays an important role in the development of the stereoscopic anisotropy.^13^ In that study, the authors measured the stereoscopic anisotropy of 159 participants with ages between 3 and 73 years. Their results showed that the stereoscopic anisotropy is present in all ages, but is stronger for adults (18–73 years) than for children (3–13 years). However, they did not test whether the anisotropy changes during adulthood. If we want to compare young (between 18–45 years) and old adults (≥60 years), with their data it is difficult to extract a conclusion given that for young adults they have 67 participants but for the old group they only have 11 participants. In the present study, our objective was to compare the stereoscopic anisotropy for young and old adults. Our results show that the stereoscopic anisotropy decreases in elderly population. Interestingly, disparity thresholds for vertical corrugations are constant across the adulthood, thus, the reduction of the anisotropy is caused by an increase of the disparity thresholds for the horizontal corrugations. This effect is the opposite of the one driving the differences found between the anisotropy in children and adults, where disparity thresholds for vertical corrugations were lower in children.^13^

## Methods

For each participant, we measured the stereoacuity and disparity thresholds for detecting vertical and horizontal sinusoidal corrugations.

### Subjects

**Figure 1.**
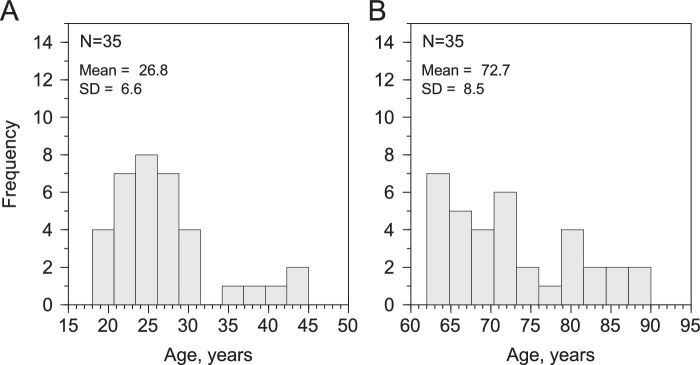
Histograms of age for the young group (**A**) and the old group (**B**). The mean (in years) and the standard deviation are located in the top left corner of the plots.

A total of 70 participants performed the experiment, 35 (17 females) for the young group (mean, 26.8 ± 6.6 years), and 35 (28 females) for the old group (mean, 72.7 ± 8.5 years). The distribution of ages for both groups can be seen in Figure 1. All participants reported having normal or corrected to normal visual acuity. All participants were tested in a dark room at their homes. We decided to test all participants this way given the difficulties of bringing old participants to the laboratory and given that the testing equipment was very easy to setup. All participants were tested with the same equipment.

Before starting the experiment, we measured the stereoacuity of all participants with the Randot Stereotest using the Graded Circle test (Stereo Optical Company, Inc., Chicago, IL, USA; www.stereooptical.com/products/stereotests-color-tests/randot/). We tested the participants at 40 cm. Participants were given an instruction sheet to read before completing the experiment and provided informed written consent. The study protocol was compliant with the Declaration of Helsinki and was approved by the Ethics Committee of the Complutense University of Madrid.

### Apparatus

The stimuli were presented on a 23-inch LG 3D monitor (D2342, LG, Yeouido-dong, Seoul, South Korea) with a screen size of 51 cm × 28.7 cm, subtending an angle of 28.61° × 16.33°. This is a passive 3D monitor, where circular polarization is used to separate the left and right images (i.e., odd rows go through one filter and even rows go through the other filter). Observers wore the passive LG 3D glasses (LG, Yeouido-dong, Seoul, South Korea) that are appropriate for the monitor (the interocular cross-talk was <2%). The spatial resolution of the monitor was 1920 × 1080 pixels (1920 × 540 pixels to each eye), and the frame rate was 60 Hz. Observers sat at a viewing distance of 100 cm so a pixel in the center of the screen subtended 53.65 seconds of arc (arcsec). We did not use a head rest, but the head position and distance were closely monitored by the experimenter. Participants recorded their responses by pressing a red or a green switch (3.5 cm activation surface, AbleNet, Inc., Roseville, MN, USA). The experiments were conducted using MacBook Air computer (13-inch, 2017, 1.8 GHz Intel Core i5), with an Intel HD Graphics 6000 (1536 MB, Santa Clara, CA, USA) graphics card, running MATLAB R2017b (The MathWorks, Inc, Natick, MA, USA).

### Stimuli

The random dot stereograms were programmed in MATLAB (The MathWorks, Inc, Natick, MA, USA) and were presented on the screen using the Psychophysics Toolbox extensions.^24^^–^^26^ The 3D image was rendered with the monitor in standard two-dimensional (2D) mode, using the line-interleaved stereo mode of Psychtoolbox's PshychImaging function and using “Screen (SelectStereoDrawBuffer)” to present the corresponding image to each eye. We generated left and right stimuli each 1000 pixels wide by 500 pixels high and interleaved them row by row to produce a single image of 1000 × 1000 pixels (subtending 15.13° × 15.13°) to send to the monitor.

**Figure 2.**
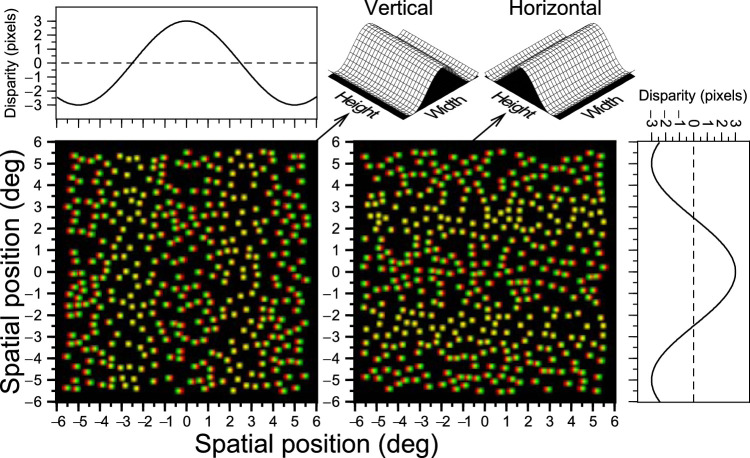
Anaglyph versions of the stimuli used in the experiment. Horizontal/vertical corrugations. The left anaglyph presents a vertical sinusoidal corrugation. The right anaglyph corresponds to a horizontal sinusoidal corrugation (for correct viewing, place the red filter in front of the left eye). The spatial frequency at the correct distance is 0.1 cycles/deg. The panels with the lines represent the disparities shown in the anaglyphs. The corresponding 3D representations are shown in the top right part of the figure. Note that this figure is for illustration purpose only, for example, the dot density is lower than the stimuli used in the experiments. Figure adapted from.^13^

The stereograms were constructed with white dots on a black background.^10^ The dots were 2D gaussians with a standard deviation of 1.816 arcmin (the gaussians occupied 7 [H] × 8 [V] [4 for odd rows and 4 for even rows] pixels) (see an example in Fig. 2). The dots were randomly located avoiding overlap with a density of 34.94 dots/deg^2^. The luminance of each pixel corresponded to the 2D Gaussian value using the central pixel as the reference position, this way we achieve subpixel disparities. The horizontal disparities on the screen (δ) to produce the sinusoidal corrugations were constructed according this equation: δ=±A/2 cos (2πfx^-φ),x^=xcos(θ)+y sin (θ); were *A* is the disparity in pixels; θ, is the orientation (0° for vertical corrugations and 90° for horizontal corrugations); φ, is the phase (it changed randomly every trial between 0 and π), and *f* is the spatial frequency (0.1 cyc/deg). The stereoscopic anisotropy appears for spatial frequencies lower than 0.4 – 1 cyc/deg.^8^^,^^10^ We have chosen 0.1 cyc/deg because this is the lowest corrugation frequency that our experimental setup is capable of displaying and because previous studies, using this spatial frequency, have found a strong stereoscopic anisotropy in adults.^8^^,^^9^^,^^13^^,^^27^^,^^28^

There is a well-known artefact that can appear when a vertically oriented sinusoidal corrugation is created using horizontal disparities.^8^^,^^10^ This artefact can produce the monocular cue of compression or expansion of the dot density. This monocular cue, that is more visible at high corrugation frequencies and high disparities, could help the subject to discriminate the vertical corrugation from a uniform dot density stimulus in a two-alternative forced choice task. Our stimuli do not present these artefacts even at a disparity of 1000 arcsec, which is much higher than the disparity thresholds for vertical corrugations obtained in our experiments.

### Procedure

Before starting the experiment, we measured the stereoacuity of the participants using the clinical test Randot Stereotest using the Graded Circle test. Then, the participants performed the main experiment. We used a two-interval forced choice task to obtain the disparity thresholds. Each trial started with a cross in the centre of the screen of 0.32° × 0.32° for 500 msec. To monitor vergence, this cross was flanked by horizontal and vertical Nonius lines of 0.7° × 0.7° so the vertical line was presented to one eye and the horizontal to the other eye. The cross was followed by 200 msec of blank screen. The presentation intervals lasted 2 seconds each. The temporal course of the trial was: cross + blank interval + first interval + cross + blank interval + second interval + participant's response. The next trial started after the subject's response. No feedback was provided about correctness. The stimulus appeared in one of the two intervals randomly. In the other interval, random dots without disparity were presented. Thus, the subject's task was to indicate in which interval the stimulus was presented. During the session, both stimuli, vertical and horizontal corrugations of 0.1 cyc/deg were randomly interleaved. In general, 20 to 25 minutes were required to obtain the disparity thresholds for both stimuli. Before starting the main experiment, all participants performed a few demonstration trials were the stimuli (vertical and horizontal sinusoidal corrugations with disparity of 316.23 arcsec) were present until the participant inform us verbally whether the corrugation was vertical or horizontal. They also performed between 5 and 10 training trials with the same procedure as in the main experiment.

To obtain the disparity thresholds for each stimulus we used Bayesian adaptive staircases.^66^ In particular, we used a procedure similar to ZEST.^29^ The probability of correct responses associated with the disparity threshold was 0.82. The details of the Bayesian staircase procedure used to estimate the disparity thresholds can be seen in^30^ (see their Supplementary Information, appendices A1 and A2). The staircase procedure had the following characteristics. (A) The prior probability–density function describing the distribution of disparity thresholds was uniform^30^^–^^32^ between x_min_ = 0.1 and x_max_ = 4.9 log_10_(arcsec) in steps of 0.001 log units. The disparity of the first trial was 316.23 arcsec (2.5 log_10_[arcsec]). (B) The model function (*M*) was the logistic function (see a complete description of this function in the Supplementary Information of,^30^ appendices A1 and A2). The spread value was 1.5, the delta parameter was 0.01, the lapse rate was 0.02, and the guess rate was 0.5. (C) The posterior distribution on the disparity threshold was updated after each trial by multiplying the prior by the model function: *M*(*x,δ*) for correct responses and 1–M(*x,δ*) for incorrect responses, where *δ* is the log-disparity just presented. The posterior of one trial was taken to be the prior of the next trial. (D) The stimulus disparity for each trial corresponded with the mean of the posterior distribution (in log-decimal values).^29^^,^^32^ (E) The staircase terminated after 30 trials.^30^ (F) The estimation of the final threshold was done by taking the mean of the final probability–density function (in log-decimal values).

When time permitted, two staircases per stimuli were run and the average of those two thresholds was taken as the final threshold. Two staircases were used in 13 participants for the young group and 13 participants for the old group; the remaining participants did only a single staircase. We have analysed the data of the participants that performed two staircases to find any practice effect. Pearson correlation between test and retest for the young group was strong for horizontal corrugations (*r* = 0.82; *P* = 0.0006; *N* = 13), and for vertical corrugations (*r* = 0.81; *P* = 0.0007; *N* = 13). A paired sample *t* test showed no significant differences between test and retest for horizontal corrugations, mean_1_ = 1.45, SD_1_ = 0.34; mean_2_ = 1.38, SD_2_ = 0.34; *t*(12) = 1.30, *P* = 0.22, and for vertical corrugations, mean_1_ = 1.97 SD_1_ = 0.35, mean_2_ = 1.96, SD_2_ = 0.41; *t*(12) = 0.12; *P* = 0.91. For the old group the test-retest correlation was also strong for horizontal corrugations (*r* = 0.83; *P* = 0.0004; *N* = 13), and mild but significant for vertical corrugations (*r* = 0.6; *P* = 0.033; *N* = 13). A paired sample *t* test showed again no significant differences between test and retest for horizontal corrugations, mean_1_ = 1.62, SD_1_ = 0.46; mean_2_ = 1.67, SD_2_ = 0.36; *t*(12) = −0.65; *P* = 0.53, and for vertical corrugations, mean_1_ = 1.94, SD_1_ = 0.41; mean_2_ = 1.87, SD_2_ = 0.34; *t*(12) = 0.68; *P* = 0.51. Our analysis shows no significant practice effect on disparity thresholds for the subjects that performed two staircases.

## Results

Disparity thresholds are distributed roughly normally in log-space rather than linear-space.^13^^,^^30^ We accordingly perform the analysis in log-disparity throughout, except the results from the Randot Stereotest. We report the mean of disparity thresholds in log_10_(arcsec) and the geometric mean in arcsec.

### Stereoacuity as a Function of Age

**Figure 3.**
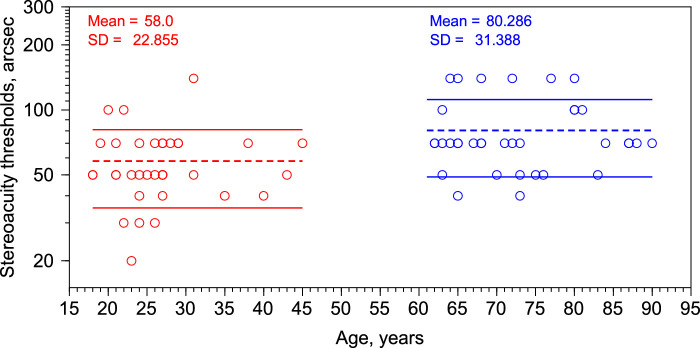
Stereoacuity thresholds (arcsec) as a function of age (years) for 70 subjects. Red dots, young group. Blue dots, old group. Dashed lines represent the mean, and continuous lines represent the mean ± 1 SD.

Figure 3 show the stereoacuity values (in arcsec) as a function of age measured using the Randot Stereotest. Red circles represent the data for the young group and blue circles for the old group. The average stereoacuity in arcsec for the young group is 58.0 ± 22.855 arcsec and for the old group 80.286 ± 31.388 arcsec (Student *t* test, *t*_2,68_ = 3.68; *P* = 0.00046 using logarithmic units; *N* = 70, with 35 participants per group). Thus, our results show that the stereoacuity thresholds, in adults, are greater with increasing age, confirming previous findings.^13^^,^^33^^,^^34^ Pearson correlation shows a significant correlation between stereoacuity thresholds (in log units) and age (*r* = 0.397; *P* = 0.0007; *N* = 70).

### Stereo Anisotropy Experiment

**Figure 4.**
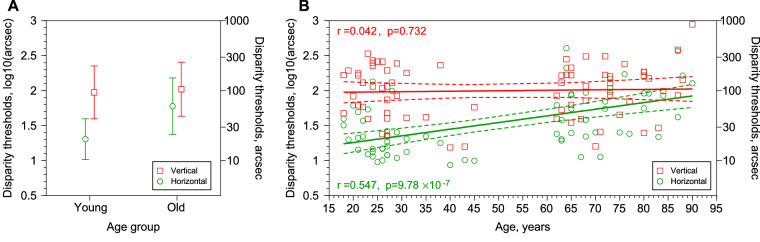
Results of the stereo anisotropy Experiment. (**A**) Disparity thresholds (mean ± 1 SD in log10[arcsec]) as a function of age group. Red squares, vertical corrugation. Green circles, horizontal corrugation. (**B**) Disparity thresholds (in log10[arcsec]) as a function of age (in years). Red squares, vertical corrugation. Green circles, horizontal corrugation. Continuous lines represent the fitted regression lines for horizontal and vertical corrugations. The fitted regression line to the disparity thresholds in logarithmic units for horizontal corrugations is Disparity_thresholds_H = 1.068 + 0.0095 × Age (years) and for vertical corrugations is Disparity_thresholds_V = 1.965 + 0.00065 × Age (years). Dashed lines are the 95% regression confidence interval.

Figure 4A shows the results of the stereo anisotropy experiment for the 70 participants. The figure shows the disparity thresholds (in log units) as a function of age. Green circles correspond with the thresholds for horizontal sinusoidal corrugations, and the red squares are the thresholds for vertical corrugations. The average for horizontal corrugations for the young group is 1.306 ± 0.292 log_10_(arcsec) (geometric mean, 20.23 arcsec) and for the old group is 1.7763 ± 0.404 log_10_(arcsec) (geometric mean, 59.74 arcsec). The average for vertical corrugations and young group is 1.9745 ± 0.379 log_10_(arcsec) (geometric mean, 94.29 arcsec) and for the old group 2.0197 ± 0.386 log_10_(arcsec) (geometric mean, 104.64 arcsec). These results replicate the classical stereoscopic anisotropy where the disparity thresholds are higher for vertical than for horizontal corrugations.^8^^,^^10^^,^^12^^,^^13^^,^^18^ Pearson correlation shows a significant correlation between disparity thresholds (in log units) of vertical and horizontal corrugations (*r* = 0.4; *P* = 0.0006; *N* = 70). Partialing out age did not change the correlation between thresholds for horizontal and vertical corrugations (*r*_partial_ = 0.45; *P* = 9.95 × 10^−5^; *N* = 70).

A mixed ANOVA with repeated measures showed significant differences between Vertical and Horizontal corrugations (within-subjects effects) (Greenhouse-Geisser *F*_1,68_ = 96.65; *P* = 1.09 × 10^−14^; partial eta squared = 0.587). We also found significant differences between age groups (between-subjects effects) (*F*_1,68_ = 11.947; *P* = 0.00095; partial eta squared = 0.149). The interaction Orientation × Age was also significant (Greenhouse-Geisser *F*_1,68_ = 21.028; *P* = 2.0 × 10^−5^; partial eta squared = 0.236). Finally, post hoc comparisons between disparity thresholds for Horizontal and Vertical corrugations showed significant differences for the young group (repeated measures *t* test, *t*_1,34_ = −8.609; *P* = 4.7 × 10^−10^) and for the old group (repeated measures *t* test, *t*_1,34_ = −4.797; *P* = 3.1 × 10^−5^).

Effect sizes comparing disparity thresholds with horizontal and vertical corrugations for each age group were calculated using the bias-corrected standardized mean difference Hedges’ *g.*^35^ We also calculated the 95% confidence interval and the biases correction for small sizes.^35^^,^^36^ We will interpret the magnitude of the Hedges’ *g* following^37^ conventions, that is, small (0.2), medium (0.5), and large (0.8). The effect size of orientation was large for the young group (Hedges’ g = −1.956; 95% CI, −2.518 to −1.384), and medium for the old group (Hedges’ g = −0.610; 95% CI, −0.964 to −0.248).

Figure 4B shows Pearson correlations and linear regressions for disparity thresholds (in log units) for horizontal and vertical corrugations as a function of age (in years). Considering that the stereoacuity thresholds increase with aging (see Fig. 3) it seems reasonable to expect that disparity thresholds for horizontal and vertical corrugations will increase with age too. The correlations show that the disparity thresholds for horizontal corrugations increase with aging (Pearson correlation coefficient: *r* = 0.547; *P* = 9.78 × 10^−7^; *N* = 70). Regressions show that for horizontal corrugations, thresholds increase with age at an average rate of 0.01 log10(arcsec) per year. However, we found no significant correlation between disparity thresholds for vertical corrugations and age (*r* = 0.042; *P* = 0.732; *N* = 70).

Comparing stereoacuity thresholds (in log units) and disparity thresholds (in log units), we found a significant correlation between stereoacuity thresholds (measured using the Randot Stereotest) and disparity thresholds for horizontal corrugations (Pearson correlation coefficient in log-thresholds: *r* = 0.32; *P* = 0.0072; *N* = 70), replicating previous results from^13^ (see their Fig. 4A). For an age range of 18 to 73 years, they^13^ found a similar correlation (*r* = 0.37; *P* = 0.0006; *N* = 84) and almost not affected after controlling the effect of age. However, with our data, the partial correlation between disparity thresholds for horizonal corrugations and stereoacuity thresholds controlling the effect of age, showed no significant correlation (*r*_partial_ = 0.132; *P* = 0.28; *N* = 70). For vertical corrugations, we found no significant correlation between stereoacuity thresholds and disparity thresholds (Pearson correlation coefficient in log thresholds: *r* = 0.12; *P* = 0.329; *N* = 70). Partialing out age did not change the correlation between stereoacuity thresholds and disparity thresholds for vertical corrugations (*r*_partial_ = 0.11; *P* = 0.364; *N* = 70). Comparing with,^13^ they found a weak correlation between stereoacuity thresholds and thresholds for vertical corrugations (see their Fig. 4B), but reanalyzing their data for an age range of 18 to 73 years, their data show no significant correlation (*r* = 0.08; *P* = 0.5; *N* = 84). We also performed Pearson correlations for our young and old groups independently, and we found no significant correlations between disparity thresholds for horizontal and vertical corrugations and stereoacuity thresholds. Thus, when controlling the effect of age, our results show no significant correlations between stereoacuity thresholds and disparity thresholds for vertical and horizontal corrugations.

**Figure 5.**
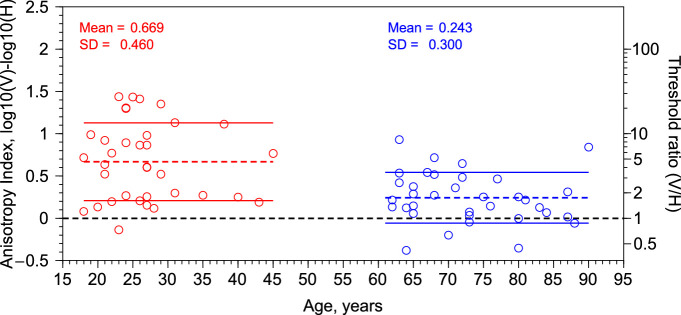
Anisotropy index as a function of age (years). Red dots, young group. Blue dots, old group. Colour dashed lines represent the mean, and continuous lines represent the mean ± 1 SD. Righthand ordinate represents the ratio between the disparity thresholds (in arcsec) of vertical corrugations and horizontal corrugations.

One way to estimate the strength of the anisotropy is to calculate an Anisotropy Index.^13^ In particular, we used the next equation AI = log_10_(V) – log_10_(H), where V and H are the disparity thresholds in arcsec. Figure 5 shows the anisotropy index as a function of age. Results show that the anisotropy is stronger for the young group (mean, 0.669 ± 0.46) than for the old group (mean, 0.243 ± 0.3). Both anisotropy indexes are significantly different from zero, one-sample *t* test: young group *t*(35) = 8.61; *P* = 4.67 × 10^−10^; old group *t*(35) = 4.797; *P* = 3.14 × 10^−5^). Student *t* test for independent samples shows significant differences between the anisotropy index of both groups (*t*_2,68_ = −4.59; *P* = 1.99 × 10^−5^). The anisotropy index is not significantly correlated with stereoacuity (Pearson correlation coefficient: *r* = −0.2; *P* = 0.09; *N* = 70) (replicating previous findings^13^). Pearson correlation between the anisotropy index and age shows a significant negative correlation (*r* = −0.49; *P* = 1.83 × 10^−5^; *N* = 70). Thus, the stereoscopic anisotropy decreases with advancing age.

## Discussion

**Figure 6.**
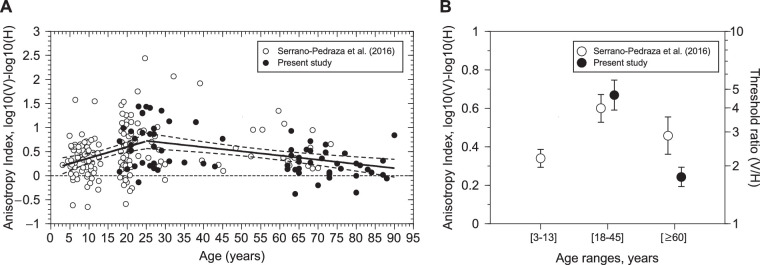
Comparison of the present study and Serrano-Pedraza et al. (2016). (**A**) Anisotropy index from S-P study (white dots) and our study (black dots) using vertical and horizontal sinusoidal corrugations of 0.1 cyc/deg (*N* = 221). Leftward fitted regression line (ages <25 years), Anisotropy_Index_Young = 0.133 + 0.023 × Age (years) (Pearson correlation: *r* = 0.297; *P* = 0.0005; *N* = 136); Rightward fitted regression line (ages ≥25 years), Anisotropy_Index_Old = 0.944–0.0088 × Age (years) (Pearson correlation: *r* = −0.39; *P* = 0.0002; *N* = 85). Dashed lines represent the 95% regression confidence interval. (**B**) Dots represent the mean ± 1 standard error of the mean. In the S-P study (white dots), in the age range of 3 to 13 years and 18 to 45 years there are 67 participants in each group, and in the age range of 60 years or more, there are only 11 participants. In the current study (black dots) we have 35 participants in the range of 18 to 45 years and 35 in the range 60 years or older. Righthand ordinate represents the ratio between the disparity thresholds (in arcsec) of vertical corrugations and horizontal corrugations. Note that each panel has a different *y* axis scaling.

In this work we have measured the stereoscopic anisotropy (i.e., higher disparity thresholds for vertical than for horizontal corrugations) in young adults (18–45 years) and old adults (62–90 years). For each participant we computed an anisotropy index, defined as the log_10_ ratio of the detection thresholds for vertical vs. horizontal corrugations. Our results clearly show, for the first time, that the anisotropy index is significantly higher in young adults than in old adults. Previous research, using different age ranges for the groups (young adults [18–32 years] and older adults [39–73 years]) found no significant differences between the anisotropy index of both groups.^13^ If we reanalyze their data for age ranges like the ones used here (young group [18–45 years], old group [≥60 years]), we have a mean anisotropy index for the young group of 0.601 ± 0.59 (*N* = 67) that is similar to our result of a mean of 0.669 ± 0.46 (*N* = 35). However, for the old group, their data show a mean anisotropy index of 0.458 ± 0.32 (*N* = 11) and we found a mean of 0.243 ± 0.3 (*N* = 35) that is almost one-half value. An independent sample *t* test shows no differences between the anisotropy indexes of both groups in the study of^13^ (*t*_2,76_ = 0.781; *P* = 0.437). Figure 6A shows the anisotropy index from both studies (in total, 221 datapoints). We have fitted two regression lines to describe the increment of the anisotropy from young age to adulthood and the decrement from adulthood to old age. In both cases the correlation is significant (see legend of the Fig. 6). Figure 6B shows a plot comparing both studies. We have also included the average of the anisotropy indexes for children (age range, 3–13 years) that is a mean of 0.34 ± 0.388 (*N* = 67).

There are two possible experimental differences that could explain the difference between our results and those of^13^ when comparing the old groups. The first one is that they have only 11 participants in this age range (≥60 years) and the second is that the oldest person in their sample had 73 years compared with us that we have 35 participants, and our oldest participant is 90 years old. Comparing the anisotropy indexes of both studies for the old groups (≥60 years) we found significant differences (*t*_2,44_ = 2.03; *P* = 0.048). Reanalyzing our data for a range of age between 60 and 73 years to match both studies, we have a mean anisotropy index of 0.284 ± 0.31 (*N* = 22) that is a bit higher than for the whole age range, but it is still significantly different from our young group (*t*_2,55_ = 3.47; *P* = 0.001). Interestingly, when the age range of the old groups is matched, there are no significant differences between the anisotropy indexes of both studies (*t*_2,31_ = 1.51; *P* = 0.142). Therefore, the differences between both studies are consistent with a different age range. Thus, the stereoscopic anisotropy increases during development from young age to adulthood and then it decreases to old age.

### Stereoacuity and the Stereoscopic Anisotropy

Previous studies have reported an improvement in stereoacuity until approximately 10 years of age,^38^^–^^42^ then no change until approximately the age of 50 years, and thereafter a decline.^33^^,^^34^^,^^43^^,^^44^ Our results are consistent with previous studies that showed a decline in stereoacuity with aging. According to the results presented here (see Fig. 5), the strength of the anisotropy could be related to the stereoacuity, that is, lower stereoacuity (e.g., in children and elderly) corresponds with lower anisotropy, but our results showed a nonsignificant correlation between anisotropy index and the stereoacuity thresholds. A general decrease in the stereoacuity in children and old adults would be expected to produce higher thresholds for horizontal, but also for vertical corrugations and therefore no changes in the anisotropy should be found; however, our results showed that when controlling the effect of age, there is no significant correlation between stereoacuity thresholds and disparity thresholds for vertical and horizontal corrugations. Serrano-Pedraza et al.^13^ showed that the reduction of the anisotropy in children was given mainly by the disparity thresholds to vertical corrugations. They found that small children presented lower disparity thresholds for vertical corrugations than adults. Given that children also presented higher thresholds for horizontal corrugations than adults, then, the anisotropy index was smaller than for adults. In our study, we have found that the disparity thresholds for horizontal corrugations are higher for the old group; however, we have found a surprising result, namely that disparity thresholds for vertical depth corrugations are constant during adulthood. As a result, the strength of the anisotropy for old adults is smaller than for young adults and similar to the anisotropy of small children (see Fig. 6B).

There are other visual aspects that could be related with the reduction of the anisotropy, for example, the well-known decrease in visual acuity with aging^45^ and the link between visual acuity and stereoacuity.^46^ However, the same logic applies here: a lower acuity would be expected to affect both orientations equally given that our stimuli are composed of isotropic luminance gaussian dots, and the vertical and horizontal corrugations differ only in the horizontal disparities across the visual field. The same argument also applies to other visual problems such uncorrected refractive errors or amblyopia.

To summarize, for observers older than 60 years, we found an increment of disparity thresholds for detecting horizontal depth corrugations (i.e., decrease in sensitivity); however, disparity thresholds for detecting vertical corrugations are not affected by aging. This could mean that the sensitivity to surfaces rotated around the horizontal axis (i.e., inclined surfaces that consist of shear disparities) is reduced in old adults but the sensitivity to surfaces rotated around the vertical axis (i.e., slanted surfaces that consist of compression disparities) is not affected across adulthood.

### Reasons for the Stereoscopic Anisotropy

As discussed in previous research,^13^ there are no clear reasons for the stereoscopic anisotropy. In the luminance domain, there is a different orientation anisotropy that could help us to understand the stereoscopic anisotropy, namely, humans have more sensitivity (i.e., higher visual acuities) to horizontal and vertical orientations than to oblique orientations (the oblique effect).^47^^–^^49^ One of the hypotheses has suggested an ecological component where the predominance of horizontal and vertical contours and angles in natural images could explain this oblique effect.^50^^–^^53^ In general, the amount of horizontal and vertical content is higher than the oblique content, however, the authors have also found different orientation bias regarding horizontal and vertical content.^50^^–^^53^ Interestingly, when using broad-band oriented stimuli, the visual sensitivity was higher for oblique than for horizontal or vertical orientations.^54^ Thus, although there is an overrepresentation of visual neurons tuned to cardinal orientations,^55^^–^^57^ and the responses of the primary visual cortex to oriented stimuli in humans are stronger to vertical and horizontal orientations than for oblique stimuli,^58^ the hypotheses that the visual system develops to match the most predominant orientation is not clear. In other words, for simple stimuli the visual system has higher sensitivity to the most predominant orientations (e.g., horizontal and vertical), but for naturalistic stimuli, it seems that the visual system may discount the most prevalent orientations to accommodate the anisotropy present in natural scenes.^53^ Regarding stereopsis, recent studies have shown that neural processes reflect statistical properties of the visual scene.^59^^–^^62^ For example, the distribution of natural disparities is consistent with the disparity preferences in the macaque visual cortex,^61^ or that rapid binocular eye movements are adapted to the statistics of the 3D environment.^60^ The matching between neuronal processes and the statistical properties poses an important question, are these neuronal processes genetically hard-wired or depend on visual experience? There are examples that are inconsistent with learning (e.g., positions of corresponding points do not change after 1 week of exposure to distorted binocular disparities),^63^ but there are also examples that show the importance of visual experience.^64^^,^^65^ The results from Serrano-Pedraza et al.^13^ showed that the stereoscopic anisotropy strengthens during development, suggesting that this anisotropy depends on visual experience. Thus, one potential reason for this anisotropy is that it reflects the statistical properties of the visual scene. Given that the anisotropy is found mainly at low spatial frequencies, the statistics of the 3D environment should show more power at horizontal than vertical corrugations at low spatial frequencies and similar power for horizontal and vertical corrugations at high spatial frequencies. So far, no evidence about this difference has been shown. The fact that disparity thresholds for horizontal corrugations increase with aging and that disparity thresholds for vertical corrugations do not change, cannot be explained by the statistics of the 3D environment. Our new results show that the anisotropy is reduced by aging, but only because the sensitivity to horizontal depth corrugations is affected.

## Conclusions

We have provided evidence that the stereoscopic anisotropy is reduced in elderly population. Disparity thresholds for low-spatial frequency horizontal depth corrugations decrease from childhood, they are almost constant during adulthood, and they increase with aging. In contrast, disparity thresholds for low spatial frequency vertical depth corrugations increase from childhood and keep constant across adulthood and aging. Thus, the reduction of the anisotropy in aging is caused by an increase of the disparity thresholds for horizontal depth corrugations.
